# Supplementary figures and images for: Measuring coral calcification under ocean acidification: methodological considerations for the 45Ca-uptake and total alkalinity anomaly technique
Source: PeerJ. 2017 Sep 1;5:e3749. doi: 10.7717/peerj.3749 (PMC5582612; doi:10.7717/peerj.3749)

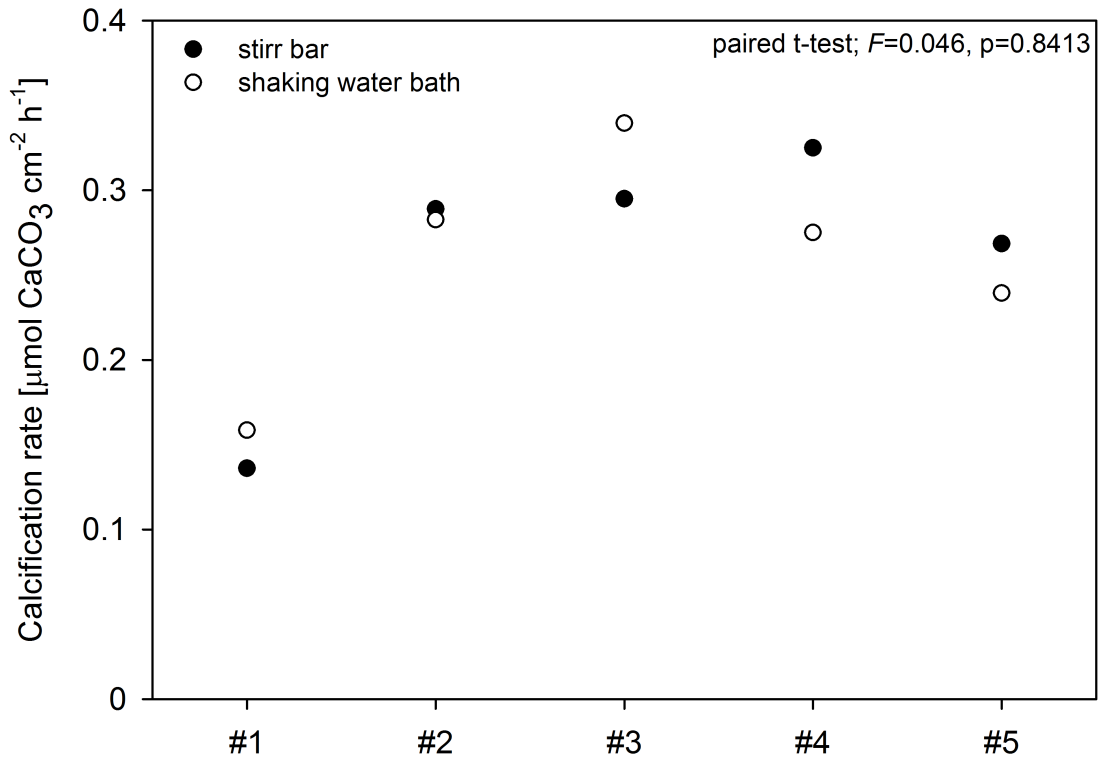

Supplement: Figure S1 — Calcification rates of five Stylophora pistillata microcolonies under ambient pH conditions as measured by the total alkalinity technique. Comparison of calcification rates derived from stirring (magnetic stirrer) or shaking (water bath) 40 mL incubation chambers over 1 h of incubation. Both methods are widely used in the literature for generating water motion inside an incubation chamber. Experiments were conducted over two consecutive days using the same fragments and results tested as pairwise t-test. [file peerj-05-3749-s001.pdf]

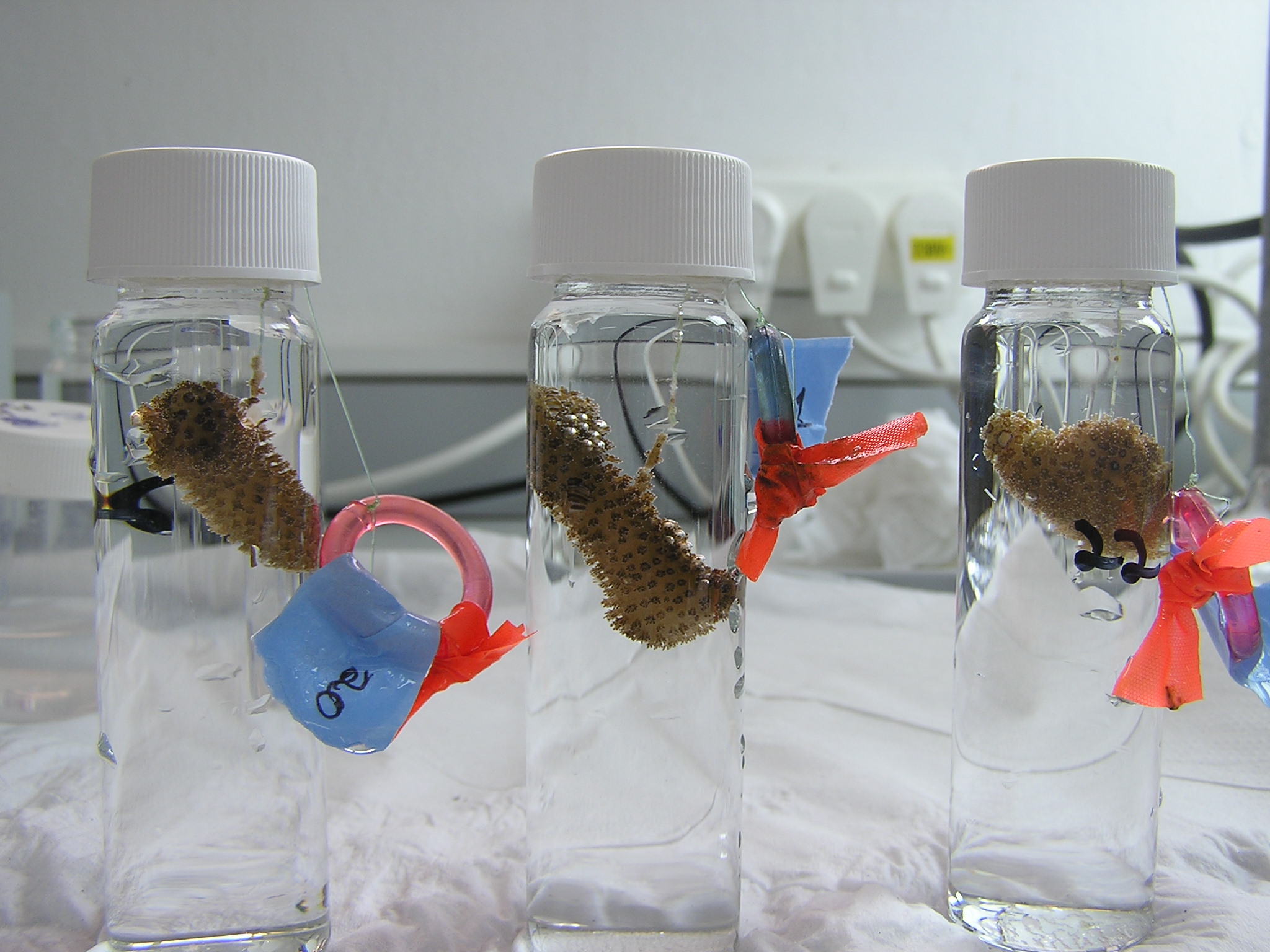

Supplement: Figure S2 — Microcolonies were fully covered by tissue and were suspended on nylon threads. Note that corals did not touch the wall of the vials. [file peerj-05-3749-s002.jpg]

A

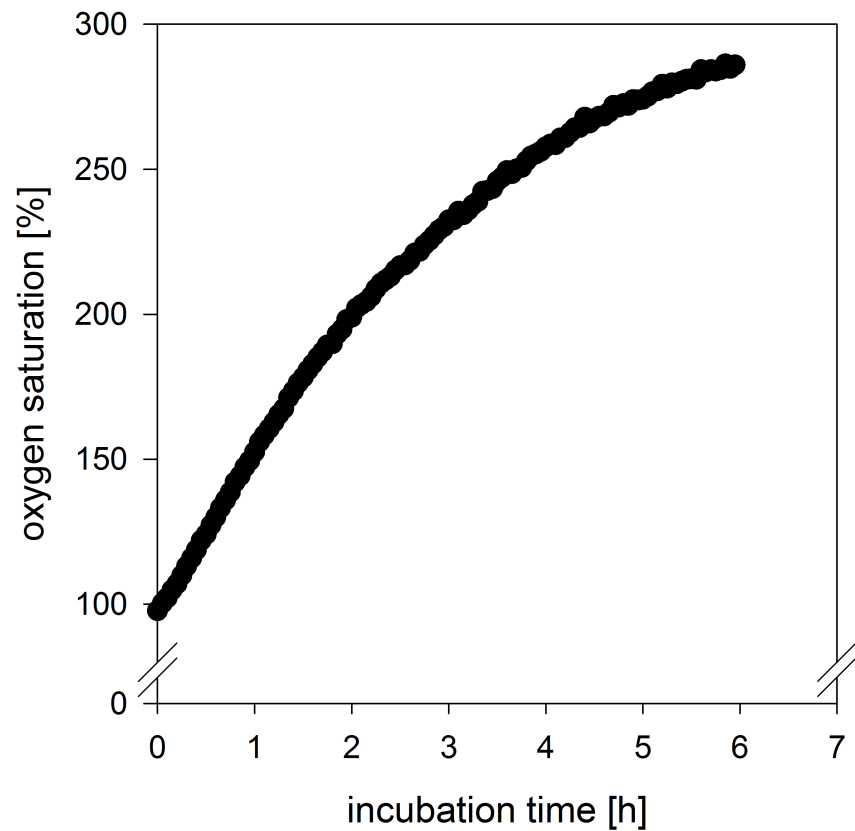

B

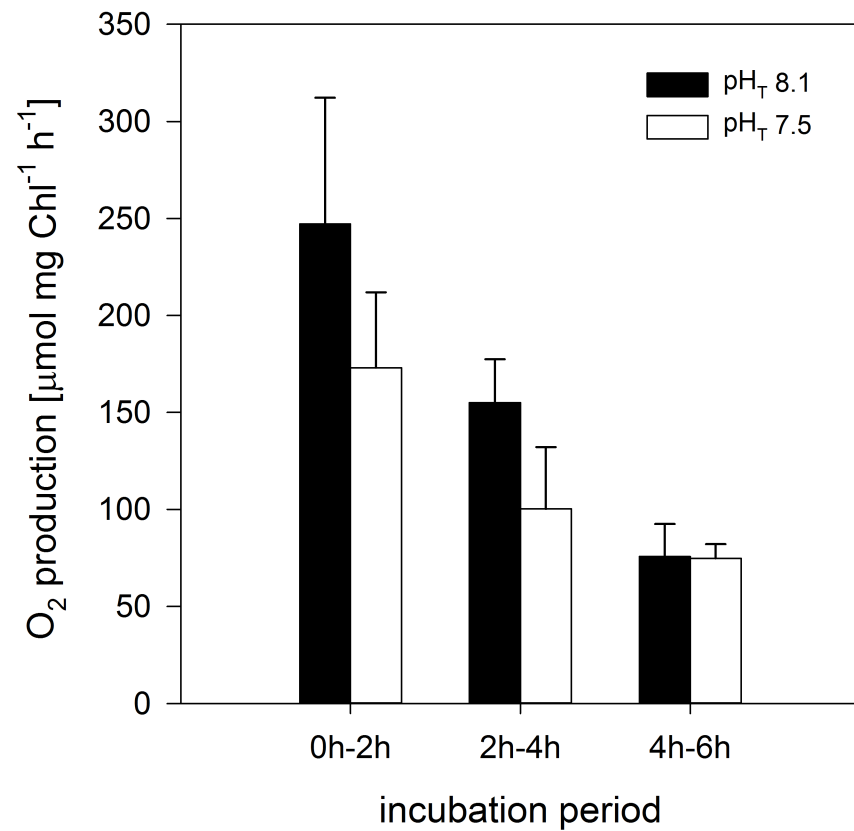

Supplement: Figure S3 — (A) Representative graph for general oxygen saturation levels in 40 mL incubation vials over time. 100% saturation corresponds to an oxygen content of 6.581 mg L−1 for the used seawater. (B) Corresponding coral O2-production rates for the three phases of the incubation period at ambient and reduced pH. Means ±SD, N = 3. Two-Way ANOVA; effect of incubation interval F2,16 = 19.918, p < 0.0001; effect of pH F1,17 = 6.035, p = 0.0277; no interaction. [file peerj-05-3749-s003.pdf]
