# Supplementary material for: Measuring coral calcification under ocean acidification: methodological considerations for the 45Ca-uptake and total alkalinity anomaly technique
Source: PeerJ. 2017 Sep 1;5:e3749. doi: 10.7717/peerj.3749 (PMC5582612; doi:10.7717/peerj.3749)
Supplement: Table S1 — The table contains information on tropical reef-building corals only. Please note that for some studies level of response to seawater acidification was affected by an additional environmental factor such as nutrients, light intensity, etc. (detailed in ‘Notes’ column). Incubation time and incubation volume are only relevant for experiments that the TA method was used to derive net calcification rates. 0 value, calcification rates were not significantly different from ambient pH; TA, total alkalinity; BW, buoyant weight. [file peerj-05-3749-s004.pdf]

Table S1. Summary of the available experimental data examining the effect of ocean acidification conditions on coral calcification. The table contains information on tropical reef-building corals only. Please note that for some studies level of response to seawater acidification was affected by an additional environmental factor such as nutrients, light intensity, etc. (detailed in `Notes` column). Incubation time and incubation volume are only relevant for experiments that the TA method was used to derive net calcification rates. 0 value, calcification rates were not significantly different from ambient pH; TA, total alkalinity; BW, buoyant weight.

Organism/ System	Approx. % change in calcification when pCO_2 is								
	~1-2x preindustrial	~3-5x preindustrial	References	Method	Acclimation time	Incubation time	Incubation volume	Coral size	Notes
Acropora cervicornis	-9		Renegar and Riegl 2005	BW	N/A	16 weeks			Response described here only for period 1
Acropora cervicornis		45	Enochs et al. 2014	BW	9 days	42 days			Level of response is light-dependent
Acoropora digitifera	0	0	Takahashi and Kurihara 2013	TA and BW	7-35 days	2 h (for TA only. Repeated 3 times through the experiment)	0.45 L	2 cm long	
Acropora eurystoma	-55		Schneider and Erez 2006	TA	N/A	2 h	0.7 L	no information on coral size	Each experiment one of the carbonate parameter kept constant
Acropora hyacinthus	8	-26	Comeau et al. 2014d	BW	6 weeks	N/A			
Acropora horrida	40		Suggett et al. 2013	TA	6 weeks	4 h	0.75 L	5-8 cm long (2 nubbins in each chambers)	Level of response is light-dependent
Acropora intermedia	14	10	Anthony et al. 2008	BW	N/A	8 weeks			Level of response is temperature-dependent
Acropora millepora	0-(-155)		Vogel et al. 2015	TA and BW	16 days	1 h	0.2 L	no information on coral size	Level of response is light-dependent
Acropora millepora	-44		Strahl et al. 2015	TA	Natural environment	1.6-2.5 h	0.64 L	5-6 cm long (3-5 cm^2)	
Acropora nasuta	0-(-)29	27	Huang et al. 2014	BW	No acclimation	94 days			

Organism/ System	Approx. % change in calcification when pCO_2 is								
	~1-2x preindustrial	~3-5x preindustrial	References	Method	Acclimation time	Incubation time	Incubation volume	Coral size	Notes
Acropora pulchra	0	0-(-14)	Comeau et al. 2014b	BW	N/A	20 days			Level of response is light-dependent
Acropora verweyi	-18		Marubini et al. 2003	BW	N/A	8 days			
Fungia sp.	-42		Hossain and Ohde 2006	TA	N/A	3-6 h	~0.5-1 L	7.1-39.5 cm^2 (1.5-3.5 cm radius)	
Galaxea fascicularis	-16		Marubini et al. 2003	BW	N/A	8 days			
Montipora capitata	5		Jokiel et al. 2008	BW	N/A	11 months			BW was measured periodically
Madracis auretenra	0-(+16)		Jury et al. 2010	TA	3 h	2 h	0.25 L	3 cm long	Level of response is depend on SW chemistry
Pavona cactus	-18		Marubini et al. 2003	BW	N/A	8 days			
Pocillopora damicornis	0		Comeau et al. 2013b	BW	N/A	3 weeks			
Pocillopora damicornis	40	48	Huang et al. 2014	BW	No acclimation	94 days			
Pocillopora damicornis	0		Strahl et al. 2015	TA	Natural environment	1.6-2.5 h	0.64 L	5-6 cm long (3-5 cm^2)	
Pocillopora damicornis	0	0	Comeau et al. 2014a	BW	N/A	14 days			
Porites astreoides	-30	-66	Crook et al. 2013	Linear extension and skeleton density (CT of skeletal core)	Natural environment	N/A			
Porites australiensis		3	Iguchi at el. 2012	BW	N/A	8 weeks			

Organism/ System	Approx. % change in calcification when pCO_2 is								
	~1-2x preindustrial	~3-5x preindustrial	References	Method	Acclimation time	Incubation time	Incubation volume	Coral size	Notes
Porites compressa	19		Marubini et al. 2001	BW	N/A	6 weeks			
Porites cylindrica	30		Suggett et al. 2013	TA	7 weeks	5 h	0.75 L	5-8 cm long (2 nubbins in each chambers)	
Porites furcata	-69	-94	Stubler et al. 2014	BW	N/A	51 days			
Porites lobata	-35	10	Anthony et al. 2008	BW	N/A	8 weeks			Level of response is temperature-dependent
Porites lutea	-42		Hossain and Ohde 2006	TA	N/A	3-6 h	~0.5-1 L	40-43 cm^2 (7.1-7.8 cm radius)	
Porites lutea	69		Ohde and Hossian 2004	TA	No acclimation	3-6 h	~0.5-1 L	48-211 cm^2 (3.8-4.5 cm radius)	
Porites rus	0		Comeau et al. 2013a	BW	3 weeks	1 week			Level of response was examined at different light and feeding regime
Porites spp.	0		Strahl et al. 2015	TA	Natural environment	1.6-2.5 h	0.64 L	5-6 cm long (3-5 cm^2)	
Porites sp.		20	Krief et al. 2010	Lateral growth	N/A	13 months			
Porites spp.	0	0	Edmunds 2012	BW	N/A	11 days			
Porites spp.	0-(+40)		Edmunds 2011	BW	N/A	1 month			Level of response depend on feeding regime
Porites spp.	0	0	Comeau et al. 2014a	BW	N/A	14 days			

Organism/ System	Approx. % change in calcification when pCO_2 is								
	~1-2x preindustrial	~3-5x preindustrial	References	Method	Acclimation time	Incubation time	Incubation volume	Coral size	Notes
Stylophora pistillata	0	-14	Gatusso et al. 1998	TA	20 days	2.5 h	0.25 L	3-5 cm long	
Stylophora pistillata	55		Reynaud et al. 2003	BW	N/A	5 weeks			Level of response is temperature-dependent
Stylophora pistillata	0-(-15)	9	Marubini et al. 2008	BW	N/A	8 days			
Stylophora pistillata	0	0	Houlbreque et al. 2012	^{45}Ca uptake	3-6 weeks	7 h	7 L	2 cm long (3 corals in each tank)	
Stylophora pistillata		21	Houlbreque et al. 2015	^{45}Ca uptake	5 weeks	7 h	7 L	5 cm long (5 corals in each tank)	Level of response is feeding-dependent
Stylophora pistillata		42	Krief et al. 2010	Lateral growth and BW	N/A	3 months			
Stylophora pistillata	7		Gagnon et al. 2013	TA and ^{43}Ca uptake-bulk measurements	N/A	5.5 days	0.215 L (flow-through chambers)	1-2 cm long	Water samples for TA were taken every ~10h
Stylophora pistillata	3	-5	Venn et al. 2013	Cross-sectional and lateral growth	> 1 year	4 h for cross-sectional growth and 2 months for lateral growth			
Stylophora pistillata	0	2	Tambutte et al. 2015	BW and skeleton density	>1 year	N/A			
Stylophora pistillata	0	0-(-50)	Holcomb et al. 2014	Cross-sectional and lateral growth	>1 year	4 h for cross-sectional growth and 2 months for lateral growth			
Siderastrea siderea	31	0	Castillo et al. 2014	BW	30-90 days	N/A			

Organism/ System	Approx. % change in calcification when pCO_2 is		References	Method	Acclimation time	Incubation time	Incubation volume	Coral size	Notes
	~1-2x preindustrial	~3-5x preindustrial							
Seriatopora hystrix		-105	Rädecker et al. 2014	TA	20 h	< 2 h	0.25 L	11.75±1.12 cm^2	
Seriatopora hystrix	-38		Strahl et al. 2015	TA	Natural environment	1.6-2.5 h	0.64 L	5-6 cm long (3-5 cm^2)	
Turbinaria reniformis	-13		Marubini et al. 2003	BW	N/A	8 days			
Porites lobata and P. lutea	0		Wall and Edmunds. 2014	TA	No acclimation	4 h	2.47 L	4 cm long	
Siderastrea radians, Solenastrea, hyades	2		Okazaki et al. 2013	TA	Natural environment	1.5 h	2 L	45±17 cm^2 and 138±43 cm^2 respectively	
Psammocora profundacella, Pocillopora verrucosa, Pocillopora damicornis, Pavona cactus	0-(-8%)	~(-17)	Comeau et al. 2014c	BW	N/A	2 weeks			divided in fast and slow growing corals
massive Porites spp., Porites rus, Acropora pulchra, Porites irregularis	0-(-17)	~(-19)	Comeau et al. 2014c	BW	N/A	2 weeks			divided in fast and slow growing corals
Acropora pulchra, Pavona cactusand Porites rus	-9		Comeau et al. 2013b	BW	N/A	2 weeks			
Porites australiensis, Isopora palifera	-48(-52)	-66(-128)	Iguchi at el. 2014	BW	N/A	43 and 30 days, respectively			
Porites compressa + Montipora capitata	43		Langdon and Atkinson 2005	TA	2 weeks	1.5 h	Flume area: 3.84 m^3	Community cover: 2.2 m^3	Level of response varies seasonally

References:

Anthony, K. R. N., D. I. Kline, G. Diaz-Pulido, S. Dove, and O. Hoegh-Guldberg. 2008. Ocean acidification causes bleaching and productivity loss in coral reef builders. Proc. Natl. Acad. Sci. U. S. A. 105: 17442-17446.

Castillo, K. D., J. B. Ries, J. F. Bruno, and I. T. Westfield. 2014. The reef-building coral *Siderastrea siderea* exhibits parabolic responses to ocean acidification and warming. P. Roy. Soc. Lond. B. Bio. 281: 20141856.

Comeau, S., R. Carpenter, Y. Nojiri, H. Putnam, K. Sakai, and P. Edmunds. 2014a. Pacific-wide contrast highlights resistance of reef calcifiers to ocean acidification. P. Roy. Soc. Lond. B. Bio. 281: 20141339.

Comeau, S., R. C. Carpenter, and P. J. Edmunds. 2013a. Effects of feeding and light intensity on the response of the coral *Porites rus* to ocean acidification. Mar. biol. 160: 1127-1134.

---. 2014b. Effects of irradiance on the response of the coral *Acropora pulchra* and the calcifying alga *Hydrolithon reinboldii* to temperature elevation and ocean acidification. J. Exp. Mar. Biol. Ecol. 453: 28-35.

Comeau, S., P. Edmunds, N. Spindel, and R. Carpenter. 2014c. Fast coral reef calcifiers are more sensitive to ocean acidification in short-term laboratory incubations. Limnol. Oceanogr. 59: 1081-1091.

Comeau, S., P. J. Edmunds, N. Spindel, and R. C. Carpenter. 2013b. The responses of eight coral reef calcifiers to increasing partial pressure of CO_2 do not exhibit a tipping point. Limnol. Oceanogr. 58: 388-398.

Comeau, S., P. J. Edmunds, N. B. Spindel, and R. C. Carpenter. 2014d. Diel pCO_2 oscillations modulate the response of the coral *Acropora hyacinthus* to ocean acidification. Mar. Ecol. Prog. Ser. 501: 99-111.

Crook, E. D., A. L. Cohen, M. Rebolledo-Vieyra, L. Hernandez, and A. Paytan. 2013. Reduced calcification and lack of acclimatization by coral colonies growing in areas of persistent natural acidification. Proc. Natl. Acad. Sci. 110: 11044-11049.

Edmunds, P. J. 2011. Zooplanktivory ameliorates the effects of ocean acidification on the reef coral *Porites spp*. Limnol. Oceanogr . 56: 2402-2410.

---. 2012. Effect of pCO_2 on the growth, respiration, and photophysiology of massive *Porites spp*. in Moorea, French Polynesia. Mar. Biol. 159: 2149-2160.

Edmunds, P. J., D. Brown, and V. Moriarty. 2012. Interactive effects of ocean acidification and temperature on two scleractinian corals from Moorea, French Polynesia. Glob. Change Biol. 18: 2173-2183.

Enochs, I., D. Manzello, R. Carlton, S. Schopmeyer, R. Van Hooidonk, and D. Lirman. 2014. Effects of light and elevated pCO2 on the growth and photochemical efficiency of *Acropora cervicornis*. Coral Reefs 33: 477-485.

Gagnon, A. C., J. F. Adkins, J. Erez, J. M. Eiler, and Y. Guan. 2013. Sr/Ca sensitivity to aragonite saturation state in cultured subsamples from a single colony of coral: Mechanism of biomineralization during ocean acidification. Geochim. Cosmochim. Ac. 105: 240-254.

Gattuso, J.-P., M. Frankignoulle, I. Bourge, S. Romaine, and R. Buddemeier. 1998. Effect of calcium carbonate saturation of seawater on coral calcification. Glob. Plant. Change 18: 37-46.

Holcomb, M. and others 2014. Coral calcifying fluid pH dictates response to ocean acidification. Sci. Rep. 4.

Hossain, M. M. M., and S. Ohde. 2006. Calcification of cultured *Porites* and *Fungia* under different aragonite saturation states of seawater. p. 597-606. Proceedings of the 10th International Coral Reef Symposium, Okinawa, Japan, 28 June-2 July 2004.

Houlbrèque, F., S. Reynaud, C. Godinot, F. Oberhänsli, R. Rodolfo-Metalpa, and C. Ferrier-Pagès. 2015. Ocean acidification reduces feeding rates in the scleractinian coral *Stylophora pistillata*. Limnol. Oceanogr. 60: 89-99.

Houlbrèque, F. and others 2012. Effects of increased pCO2 on zinc uptake and calcification in the tropical coral *Stylophora pistillata*. Coral Reefs 31: 101-109.

Huang, H., X.-C. Yuan, W.-J. Cai, C.-L. Zhang, X. Li, and S. Liu. 2014. Positive and negative responses of coral calcification to elevated pCO_2: case studies of two coral species and the implications of their responses. Mar. Ecol. Prog. Ser. 502: 145.

Iguchi, A., N. H. Kumagai, T. Nakamura, A. Suzuki, K. Sakai, and Y. Nojiri. 2014. Responses of calcification of massive and encrusting corals to past, present, and near-future ocean carbon dioxide concentrations. Mar. Pollut. Bull. 89: 348-355.

Iguchi, A. and others 2012. Effects of acidified seawater on coral calcification and symbiotic algae on the massive coral *Porites australiensis*. Mar. Environ. Res. 73: 32-36.

Jokiel, P. L., K. S. Rodgers, I. B. Kuffner, A. J. Andersson, E. F. Cox, and F. T. Mackenzie. 2008. Ocean acidification and calcifying reef organisms: a mesocosm investigation. Coral Reefs 27: 473-483.

Jury, C. P., R. F. Whitehead, and A. M. Szmant. 2010. Effects of variations in carbonate chemistry on the calcification rates of *Madracis auretenra* (= *Madracis mirabilis* sensu Wells, 1973): bicarbonate concentrations best predict calcification rates. Glob.
Change Biol. 16: 1632-1644.

Krief, S. and others 2010. Physiological and isotopic responses of scleractinian corals to ocean acidification. Geochim. Cosmochim. Ac. 74: 4988-5001.

Langdon, C., and M. J. Atkinson. 2005. Effect of elevated pCO_2 on photosynthesis and calcification of corals and interactions with seasonal change in temperature/irradiance and nutrient enrichment. J. Geophys. Res-Oceans 110.

Marubini, F., H. Barnett, C. Langdon, and M. Atkinson. 2001. Dependence of calcification on light and carbonate ion concentration for the hermatypic coral *Porites compressa*. Mar. Ecol. Prog. Ser. 220: 153-162.

Marubini, F., C. Ferrier-Pagès, P. Furla, and D. Allemand. 2008. Coral calcification responds to seawater acidification: a working hypothesis towards a physiological mechanism. Coral Reefs 27: 491-499.

Marubini, F., C. Ferrier–Pages, and J. P. Cuif. 2003. Suppression of skeletal growth in scleractinian corals by decreasing ambient carbonate-ion concentration: a cross-family comparison. P. Roy. Soc. Lond. B. Bio. 270: 179-184.

Ohde, S., and M. M. M. Hossain. 2004. Effect of $CaCO_3$ (aragonite) saturation state of seawater on calcification of *Porites* coral. Geochem. J. 38: 613-621.

Okazaki, R., P. Swart, and C. Langdon. 2013. Stress-tolerant corals of Florida Bay are vulnerable to ocean acidification. Coral Reefs 32: 671-683.

Rädecker, N., F. W. Meyer, V. N. Bednarz, U. Cardini, and C. Wild. 2014. Ocean acidification rapidly reduces dinitrogen fixation associated with the hermatypic coral *Seriatopora hystrix*. Mar. Ecol. Prog. Ser. 511: 297.
Renegar, D. A., and B. M. Riegl. 2005. Effect of nutrient enrichment and elevated CO_2 partial pressure on growth rate of Atlantic scleractinian coral *Acropora cervicornis*. Mar. Ecol. Prog. Ser. 293: 69-76.

Reynaud, S., N. Leclercq, S. Romaine-Lioud, C. Ferrier-Pages, J. Jaubert, and J. P. Gattuso. 2003. Interacting effects of CO_2 partial pressure and temperature on photosynthesis and calcification in a scleractinian coral. Glob. Change Biol. 9: 1660-1668.

Schneider, K., and J. Erez. 2006. The effect of carbonate chemistry on calcification and photosynthesis in the hermatypic coral *Acropora eurystoma*. Limnol. Oceanogr. 51: 1284-1293.

Strahl, J., I. Stolz, S. Uthicke, N. Vogel, S. Noonan, and K. Fabricius. 2015. Physiological and ecological performance differs in four coral taxa at a volcanic carbon dioxide seep. Comp. Biochem. Phys. A. 184: 179-186.

Stubler, A. D., B. T. Furman, and B. J. Peterson. 2014. Effects of pCO_2 on the interaction between an excavating sponge, *Cliona varian*s, and a hermatypic coral, *Porites furcata*. Mar. Biol. 161: 1851-1859.

Suggett, D. J., L. F. Dong, T. Lawson, E. Lawrenz, L. Torres, and D. J. Smith. 2013. Light availability determines susceptibility of reef building corals to ocean acidification. Coral Reefs 32: 327-337.

Takahashi, A., and H. Kurihara. 2013. Ocean acidification does not affect the physiology of the tropical coral *Acropora digitifera* during a 5-week experiment. Coral Reefs 32: 305-314.

Tambutté, E. and others 2015. Morphological plasticity of the coral skeleton under CO_2-driven seawater acidification. Nat. Commun. 6:7368.

Venn, A. A., E. Tambutté, M. Holcomb, J. Laurent, D. Allemand, and S. Tambutté. 2013. Impact of seawater acidification on pH at the tissue-skeleton interface and calcification in reef corals. Proc. Natl. Acad. Sci. 110: 1634-1639.

Vogel, N., F. Meyer, C. Wild, and S. Uthicke. 2015. Decreased light availability can amplify negative impacts of ocean acidification on calcifying coral reef organisms. Mar. Ecol. Prog. Ser. 521: 49.

Wall, C. B., and P. J. Edmunds. 2013. *In situ* effects of low pH and elevated HCO_3^- on juvenile massive *Porites spp*. in Moorea, French Polynesia. Biol. Bull. 225: 92-101.
