# Supplementary material for: Measuring coral calcification under ocean acidification: methodological considerations for the 45Ca-uptake and total alkalinity anomaly technique
Source: PeerJ. 2017 Sep 1;5:e3749. doi: 10.7717/peerj.3749 (PMC5582612; doi:10.7717/peerj.3749)
Supplement: Table S2 — Data were extracted from Fig. 2 in Smith & Kinsey (1978) and Fig. 5 in Tambutté et al. (1995), using the software WebPlotDigitizer (Rohatgi, 2015). Values from Smith & Kinsey (1978) were converted using a molecular weight of 100.0869 g/mol for CaCO3. [file peerj-05-3749-s005.docx]

**Table S2**. Raw data used to create Fig. 3 of this study. Data were extracted from Fig. 2 in Smith & Kinsey (1978) and Fig. 5 in Tambutté et al. (1995), using the software WebPlotDigitizer (Rohatgi 2015). Values from Smith & Kinsey (1978) were converted using a molecular weight of 100.0869 g/mol for CaCO_3_.

| **Smith and Kinsey (1978)** | | **Tambutté et al. (1995)** | | **this study** | | | |
| --- | --- | --- | --- | --- | --- | --- | --- |
| CaCO_3_ estimated from ^45^Ca uptake [µg] | CaCO_3_ estimated from alkalinity decrease [µg] | ^45^Ca fixation estimates [nmol] | alkalinity titration estimates [nmol] | pH | incubation time [h] | ^45^Ca fixation estimates [nmol] | alkalinity titration estimates [nmol] |
| 71 | 30 | 31 | 222 | 8.2 | 2 | 2295.4 | 2490.7 |
| 84 | 41 | 67 | 450 | 8.2 | 2 | 1211.3 | 854.1 |
| 88 | 15 | 277 | 463 | 8.2 | 2 | 2737.7 | 2275.4 |
| 93 | 5 | 228 | 463 | 8.2 | 2 | 2639.4 | 2538.1 |
| 98 | 13 | 185 | 657 | 8.2 | 2 | 1452.0 | 1653.7 |
| 103 | 13 | 348 | 721 | 8.2 | 2 | 2411.4 | 2774.2 |
| 117 | 31 | 563 | 748 | 7.5 | 2 | 2305.9 | 2930.2 |
| 124 | 34 | 522 | 853 | 7.5 | 2 | 1671.0 | 2161.6 |
| 108 | 44 | 554 | 881 | 7.5 | 2 | 2573.7 | 2805.9 |
| 118 | 50 | 471 | 1007 | 7.5 | 2 | 2869.6 | 3249.2 |
| 101 | 50 | 624 | 962 | 7.5 | 2 | 3111.7 | 3820.0 |
| 109 | 56 | 686 | 1051 | 7.5 | 2 | 2755.5 | 4122.3 |
| 135 | 63 | 784 | 1391 |  |  |  |  |
| 168 | 98 | 1245 | 1738 |  |  |  |  |
| 157 | 113 | 1340 | 2033 |  |  |  |  |
| 176 | 114 | 1550 | 2022 |  |  |  |  |
| 163 | 132 | 1632 | 1962 |  |  |  |  |
| 168 | 138 | 1836 | 2072 |  |  |  |  |
| 180 | 140 | 1751 | 2185 |  |  |  |  |
| 184 | 140 | 1723 | 2265 |  |  |  |  |
| 189 | 138 | 1643 | 2338 |  |  |  |  |
| 185 | 167 | 1982 | 2583 |  |  |  |  |
| 211 | 175 | 2255 | 2823 |  |  |  |  |
| 228 | 180 |  |  |  |  |  |  |
