# Supplementary material for: Measuring coral calcification under ocean acidification: methodological considerations for the 45Ca-uptake and total alkalinity anomaly technique
Source: PeerJ. 2017 Sep 1;5:e3749. doi: 10.7717/peerj.3749 (PMC5582612; doi:10.7717/peerj.3749)
Supplement: Table S3 — Seawater carbonate chemistry in each of the incubation vessels as recorded in the alkalinity experiment. TA and pH were measured while all other parameters were calculated using the CO2SYS program (Lewis, Wallace & Allison, 1998). [file peerj-05-3749-s006.docx]

**Table S3**. Seawater carbonate chemistry in each of the incubation vessels as recorded in the alkalinity experiment. TA and pH were measured while all other parameters were calculated using the CO2SYS program (Lewis et al. 1998).

| pH_T_ treatment | Time  [h] | Replicate number | TA | Final pH_T_ | DIC | *p*CO_2_ | CO_2(aq)_ | HCO_3_^-^ | CO_3_^2-^ | Ω_arg_ |
| --- | --- | --- | --- | --- | --- | --- | --- | --- | --- | --- |
|  |  |  | [µeq kg^-1^] |  | [µmol kg^-1^] | [µatm] | [µmol kg^-1^] | [µmol kg^-1^] | [µmol kg^-1^] |  |
| 8.1 | Control | 1 | 2507 | 8.09 | 2133 | 391 | 10.8 | 1859 | 263 | 4.01 |
|  | 2 | 1 | 2380 | 8.23 | 1920 | 244 | 6.7 | 1601 | 313 | 4.76 |
|  |  | 2 | 2463 | 8.26 | 1968 | 231 | 6.4 | 1622 | 339 | 5.17 |
|  |  | 3 | 2391 | 8.25 | 1914 | 230 | 6.4 | 1584 | 324 | 4.93 |
|  |  | 4 | 2377 | 8.29 | 1871 | 202 | 5.6 | 1524 | 342 | 5.20 |
|  |  | 5 | 2423 | 8.30 | 1901 | 200 | 5.5 | 1542 | 354 | 5.39 |
|  |  | 6 | 2367 | 8.20 | 1931 | 266 | 7.3 | 1627 | 297 | 4.52 |
|  | 4 | 1 | 2404 | 8.48 | 1734 | 110 | 3.0 | 1285 | 446 | 6.80 |
|  |  | 2 | 2402 | 8.50 | 1716 | 103 | 2.8 | 1256 | 457 | 6.96 |
|  |  | 3 | 2288 | 8.55 | 1584 | 82 | 2.3 | 1123 | 458 | 6.98 |
|  |  | 4 | 2363 | 8.54 | 1651 | 88 | 2.4 | 1178 | 470 | 7.16 |
|  |  | 5 | 2305 | 8.40 | 1723 | 137 | 3.8 | 1334 | 385 | 5.87 |
|  |  | 6 | 2361 | 8.55 | 1640 | 85 | 2.3 | 1163 | 475 | 7.23 |
|  | 6 | 1 | 2344 | 8.56 | 1619 | 81 | 2.2 | 1141 | 476 | 7.25 |
|  |  | 2 | 2337 | 8.71 | 1486 | 47 | 1.3 | 934 | 551 | 8.39 |
|  |  | 3 | 2303 | 8.58 | 1570 | 74 | 2.0 | 1091 | 477 | 7.27 |
|  |  | 4 | 2267 | 8.57 | 1551 | 76 | 2.1 | 1085 | 464 | 7.06 |
|  |  | 5 | 2280 | 8.50 | 1620 | 97 | 2.7 | 1186 | 431 | 6.57 |
|  |  | 6 | 2315 | 8.40 | 1730 | 138 | 3.8 | 1340 | 387 | 5.89 |
| 7.5 | Control | 1 | 2510 | 7.41 | 2475 | 2356 | 65.0 | 2341 | 69 | 1.05 |
|  | 2 | 1 | 2358 | 7.70 | 2211 | 1065 | 29.4 | 2063 | 119 | 1.81 |
|  |  | 2 | 2399 | 7.48 | 2338 | 1892 | 52.2 | 2209 | 77 | 1.17 |
|  |  | 3 | 2365 | 7.76 | 2191 | 913 | 25.2 | 2032 | 135 | 2.05 |
|  |  | 4 | 2343 | 7.73 | 2184 | 978 | 27.0 | 2031 | 126 | 1.91 |
|  |  | 5 | 2312 | 7.76 | 2141 | 892 | 24.6 | 1985 | 131 | 2.00 |
|  |  | 6 | 2296 | 7.77 | 2121 | 863 | 23.8 | 1964 | 133 | 2.03 |
|  | 4 | 1 | 2317 | 8.02 | 2008 | 440 | 12.1 | 1781 | 215 | 3.27 |
|  |  | 2 | 2235 | 7.90 | 2000 | 592 | 16.3 | 1818 | 166 | 2.53 |
|  |  | 3 | 2111 | 8.15 | 1741 | 273 | 7.5 | 1492 | 242 | 3.69 |
|  |  | 4 | 2239 | 8.03 | 1931 | 413 | 11.4 | 1709 | 211 | 3.21 |
|  |  | 5 | 2014 | 7.95 | 1770 | 463 | 12.8 | 1594 | 163 | 2.49 |
|  |  | 6 | 2283 | 7.96 | 2012 | 513 | 14.1 | 1808 | 190 | 2.89 |
|  | 6 | 1 | 2050 | 8.29 | 1593 | 172 | 4.7 | 1298 | 291 | 4.43 |
|  |  | 2 | 2188 | 8.34 | 1673 | 158 | 4.3 | 1333 | 336 | 5.11 |
|  |  | 3 | 2306 | 8.31 | 1795 | 184 | 5.1 | 1450 | 340 | 5.19 |
|  |  | 4 | 2054 | 8.37 | 1539 | 133 | 3.7 | 1209 | 326 | 4.97 |
|  |  | 5 | 2208 | 8.30 | 1720 | 181 | 5.0 | 1395 | 320 | 4.88 |
|  |  | 6 | 2180 | 8.18 | 1782 | 258 | 7.1 | 1512 | 263 | 4.01 |
