# Supplementary material for: Measuring coral calcification under ocean acidification: methodological considerations for the 45Ca-uptake and total alkalinity anomaly technique
Source: PeerJ. 2017 Sep 1;5:e3749. doi: 10.7717/peerj.3749 (PMC5582612; doi:10.7717/peerj.3749)
Supplement: Table S4 — Rates of alkalinity change of bare skeletons in different seawater pH treatments after 3 h of incubation. Chambers were identical to the ones used in the described experiments. The precision of the used titrator was less than 6 µeq kg−1, thus usable ΔTA values have to be at least 18 µeq kg−1. [file peerj-05-3749-s007.docx]

**Table S4**. Rates of alkalinity change of bare skeletons in different seawater pH treatments after 3 hours of incubation. Chambers were identical to the ones used in the described experiments. The precision of the used titrator was less than 6 µeq kg^-1^, thus usable ΔTA values have to be at least 18 µeq kg^-1^.

| **pH_NBS_** | **sample #** | **A_T_**  **[µeq kg^-1^]** | **∆A_T_**  **[µeq kg^-1^]** | **T**  **[hr]** | **V_chamber_ [mL]** | **V_coral_ [mL]** | **V_incubation_/V_coral_** |
| --- | --- | --- | --- | --- | --- | --- | --- |
| **8.2** | **seawater** | **2496.9** |  |  |  |  |  |
|  | 30 | 2488.1 | 8.8 | 3 | 40 | 1.22 | 32 |
|  | 31 | 2489.2 | 7.8 | 3 | 40 | 0.79 | 50 |
|  | 32 | 2492.8 | 4.1 | 3 | 40 | 0.77 | 51 |
|  | 33 | 2486.7 | 10.3 | 3 | 40 | 0.97 | 40 |
|  | 34 | 2490.0 | 6.9 | 3 | 40 | 0.98 | 40 |
|  | 35 | 2495.1 | 1.8 | 3 | 40 | 0.77 | 51 |
|  | 36 | 2489.3 | 7.7 | 3 | 40 | 1.07 | 36 |
|  | 37 | 2491.3 | 5.7 | 3 | 40 | 0.75 | 52 |
|  | 38 | 2487.9 | 9.1 | 3 | 40 | 0.99 | 39 |
| **7.6** | **seawater** | **2499.2** |  |  |  |  |  |
|  | 30 | 2499.2 | 0.0 | 3 | 40 | 1.22 | 32 |
|  | 31 | 2502.2 | -2.9 | 3 | 40 | 0.79 | 50 |
|  | 32 | 2493.4 | 5.8 | 3 | 40 | 0.77 | 51 |
|  | 33 | 2504.2 | -4.9 | 3 | 40 | 0.97 | 40 |
|  | 34 | 2490.0 | 9.2 | 3 | 40 | 0.98 | 40 |
|  | 35 | 2506.8 | -7.5 | 3 | 40 | 0.77 | 51 |
|  | 36 | 2504.6 | -5.4 | 3 | 40 | 1.07 | 36 |
|  | 37 | 2501.6 | -2.4 | 3 | 40 | 0.75 | 52 |
|  | 38 | 2495.5 | 3.7 | 3 | 40 | 0.99 | 39 |
| **7.3** | **seawater** | **2501.4** |  |  |  |  |  |
|  | 30 | 2497.3 | 4.1 | 3 | 40 | 1.22 | 32 |
|  | 31 | 2501.9 | -0.5 | 3 | 40 | 0.79 | 50 |
|  | 32 | 2498.0 | 3.5 | 3 | 40 | 0.77 | 51 |
|  | 33 | 2500.5 | 1.0 | 3 | 40 | 0.97 | 40 |
|  | 34 | 2535.3 | -33.9 | 3 | 40 | 0.98 | 40 |
|  | 35 | 2500.0 | 1.4 | 3 | 40 | 0.77 | 51 |
|  | 36 | 2524.9 | -23.4 | 3 | 40 | 1.07 | 36 |
|  | 37 | 2500.5 | 1.0 | 3 | 40 | 0.75 | 52 |
|  | 38 | 2501.8 | -0.4 | 3 | 40 | 0.99 | 39 |
