# Supplementary material for: Measuring coral calcification under ocean acidification: methodological considerations for the 45Ca-uptake and total alkalinity anomaly technique
Source: PeerJ. 2017 Sep 1;5:e3749. doi: 10.7717/peerj.3749 (PMC5582612; doi:10.7717/peerj.3749)
Supplement: Table S5 — Two-Way ANOVA results on effects of pH (pHT 8.1 vs. 7.5) and incubation time (time period 2 h, 4 h, 6 h) on changes in variables related to seawater chemistry shown in Table 4. Asterisks indicate statistical significance with N = 6. [file peerj-05-3749-s008.docx]

Table S5. Two-Way ANOVA results on effects of pH (pH_T_ 8.1 vs. 7.5) and incubation time (time period 2h, 4h, 6h) on changes in variables related to seawater chemistry shown in Table 4. Asterisks indicate statistical significance with N = 6.

|  | **Alkalinity** | **DIC** | **pH** | **CO_2(aq)_** | **HCO_3_^-^** | **CO_3_^2-^** |
| --- | --- | --- | --- | --- | --- | --- |
| pH | *F*_1,34_ = 25.939,  p < 0.0001* | *F*_1,34_ = 1.591,  p = 0.2170 | *F*_1,34_ = 9.567,  p = 0.0043* | *F*_1,34_ = 125.940,  p < 0.0001* | *F*_1,34_ = 0.086,  p = 0.7720 | *F*_1,34_ = 0.053,  p = 0.4721 |
| time | *F*_2,33_ = 11.718, p = 0.0002* | *F*_2,33_ = 74.664,  p < 0.0001* | *F*_2,33_ = 83.740,  p < 0.0001* | *F*_2,33_ = 32.019,  p < 0.0001* | *F*_2,33_ = 93.826,  p < 0.0001* | *F*_2,33_ = 72.858,  p < 0.0001* |
| pH x time | N/A | *F*_2,30_ = 4.235,  p = 0.0240* | *F*_2,30_ = 10.748,  p = 0.0003* | *F*_2,30_ = 16.533,  p < 0.0001* | *F*_2,30_ = 5.211,  p = 0.0114* | *F*_2,30_ = 6.572,  p = 0.0043* |
